# 

**Published:** 1901-06-22

**Authors:** 


### NOTICE TO CORRESPONDENTS.

**All MSS., Letters, Books for Review, and other matters intended for the Editor should be addressed The Editor, "The Hospital," The Hospital Building, 28 & 29 Southampton Street, Strand, W.C.**

**The Editor cannot undertake to return rejected MS., even when accompanied by stamped directed envelope.**

**Notice to Advertisers and Subscribers.**

**All Advertisements, Orders for Copies of The Hospital, and Business Communications should be addressed to THE MANAGER (Not to the Editor), The Hospital, 28 & 29 Southampton Street, Strand, London, W.C.**

**The Cost of Subscription, post free, is as follows:—Per week, 2½d.; per quarter, 2s. 8d.; per half-year, 5s. 3d.; per annum, 10s. 6d. Foreign:—Per annum, 15s. 2d., payable, in all cases, in advance.**

**NOTICE.—Vol. XXIX. of "The Hospital" (October 1900, to March, 1901), in handsome cloth binding is now on sale at the Office of this Journal, price, 7s. 6d. Cases for binding the Numbers contained in this Volume 1s. 6d. Index to Vol. XXIX., 2½d., post free.**

**The Monthly Part for June is now ready. Price 6d., by post 8d.**

### BOOKS RECEIVED.

W. B. Saunders and Co.

"Bacteriology and Surgical Technique for Nurses." By Emily M. A. Stoney.

Baillière, Tindall, and Cox.

"The Commonwealth of Cells." By H. G. F. Spurrell, B.A., Oxon.

Swan Sonnenschein and Co.

"An Old Friend With a New Face." By E. A. W.

"The Engineer" Office.

"Smoke and its Diminution." By Bryan Donkin, M.I.C.E., M.I.M.E.

The Cancer Society.

"An Essay on Cancer." By Alex. Frazer, M.D.

Hoyten and Cole, Plymouth.

"The Truth about the Royal Army Medical Corps. By "Justice."

"Black and White" Office.

"How to Nurse the Sick: a Handy Book of Home Nursing" (The Home Series).

James Townsend and Son.

"Thirty-one Twenty-four-hour Charts for Private Nurses." Arranged by Florence White.

R. Ackrill, Harrogate.

"The Use and Abuse of the Harrogate Mineral Waters." By Arthur Roberts, M.D.

William Heinemann.

"The Cook's Decameron." By Mrs. W. G. Waters.

T. Fisher Unwin.

"Desmonde M.D." By Henry Willard French.

### The Student of Medicine.

### APPOINTMENTS.

**Arthur Stanley Barnes**, M.B.Lond., B.Sc., appointed Junior House Physician to the National Hospital for the Paralysed and Epileptic, Queen Square. **Vernon K. Blackburn**, L.R.C.P.Lond., M.R.C.S., appointed Assistant Resident Medical Officer to the Sheffield City Hospitals for the Treatment of Cases of Infectious Diseases. **H. E. Brawn**, M.R.C.S., L.R.C.P.Lond., appointed Second Assistant Medical Officer to the St. Marylebone Infirmary. **T. W. Clay**, L.R.C.P., L.R.C.S.Edin., appointed Certifying Factory Surgeon for the Holyhead District of the County of Anglesey. **A. Coddy**, M.R.C.S., L.R.C.P.Lond., appointed Assistant Medical Officer of the Liverpool Parish Workhouse. **F. A. Cooke**, M.B., appointed District Medical Officer of the Seisdon Union. **G. Day**, L.R.C.P.&S.Edin., appointed Senior Resident, Bootle Borough Hospital. **G. F. Dickson**, M.R.C.S.Eng., L.R.C.P.Edin., appointed Medical Officer for the Potton District of the Biggleswade Union. **W. J. Dixon**, M.B., C.M.Madras, M.R.C.S.Eng., L.R.C.P.Lond., appointed Resident Medical Officer to the North-West London Hospital, Kentish Town. **W. D. Donnan**, M.D.R.U.I., B.Ch., appointed Medical Officer for the Belfast Urban (No. 14) and Castlereagh (No. 2) Dispensary District. **F. A. C. Fletcher**, L.R.C.P., L.R.C.S.Edin., appointed Certifying Factory Surgeon for the Crosshills District of the West Riding of Yorks.

